# Polariton-driven phonon laser

**DOI:** 10.1038/s41467-020-18358-z

**Published:** 2020-09-11

**Authors:** D. L. Chafatinos, A. S. Kuznetsov, S. Anguiano, A. E. Bruchhausen, A. A. Reynoso, K. Biermann, P. V. Santos, A. Fainstein

**Affiliations:** 1 Department of Physics, Centro Atómico Bariloche & Instituto Balseiro (CNEA) and CONICET, Universidad Nacional de Cuyo (UNCuyo), Av. E. Bustillo 9500, R8402AGP S.C. de Bariloche, Río Negro Argentina; 2 Paul-Drude-Institut für Festkörperelektronik, Leibniz-Institut im Forschungsverbund Berlin e.V., Hausvogteiplatz 5-7, 10117 Berlin, Germany

**Keywords:** Near-infrared spectroscopy, Bose-Einstein condensates, Semiconductors, Optomechanics, Polaritons

## Abstract

Efficient generation of phonons is an important ingredient for a prospective electrically-driven phonon laser. Hybrid quantum systems combining cavity quantum electrodynamics and optomechanics constitute a novel platform with potential for operation at the extremely high frequency range (30–300 GHz). We report on laser-like phonon emission in a hybrid system that optomechanically couples polariton Bose-Einstein condensates (BECs) with phonons in a semiconductor microcavity. The studied system comprises GaAs/AlAs quantum wells coupled to cavity-confined optical and vibrational modes. The non-resonant continuous wave laser excitation of a polariton BEC in an individual trap of a trap array, induces coherent mechanical self-oscillation, leading to the formation of spectral sidebands displaced by harmonics of the fundamental 20 GHz mode vibration frequency. This phonon “lasing” enhances the phonon occupation five orders of magnitude above the thermal value when tunable neighbor traps are red-shifted with respect to the pumped trap BEC emission at even harmonics of the vibration mode. These experiments, supported by a theoretical model, constitute the first demonstration of coherent cavity optomechanical phenomena with exciton polaritons, paving the way for new hybrid designs for quantum technologies, phonon lasers, and phonon-photon bidirectional translators.

## Abstract (web summary)

Efficient generation of phonons is an important ingredient for a prospective electrically-driven phonon laser for coherent control of quantum systems. Here, the authors report on laser-like phonon emission in a hybrid semiconductor microcavity that optomechanically couples BEC polaritons with phonons.

## Introduction

Hybrid devices composed of different physical components with complementary functionalities constitute one trending line of research for novel quantum communication technologies for signal storage, processing, conversion, and transmission^1^. Cavity optomechanics^2^ constitutes one domain, in which hybrid designs have been envisaged both for the test of fundamental quantum physics at the mesoscopic level, as well as for new functionalities^3^. In cavity optomechanics photons are confined and strongly coupled to vibrational degrees of freedom, thus leading, under appropriate conditions, to dynamical back-action phenomena, including optically induced coherent self-oscillation of the mechanical mode^4,5^ and, conversely, to laser cooling of the mechanical mode, even down to the quantum ground state^6–9^. One application of cavity optomechanics is in the bidirectional conversion between signals of contrastingly different frequency, for example, between classical microwaves and optical light^10–12^, with prospects for the transfer of quantum states^13^. Cavity polaritons, the strongly coupled quantum states combining an exciton (as a two-level artificial atom) and a cavity-confined photon, are the fundamental excitations of another hybrid quantum system^14^. Since the initial discovery of cavity polaritons in semiconductor microcavities, their Bose–Einstein condensation (BEC)^15^, superfluidity^16^, lasing^17^, also under electrical pumping^18^, and multistable behavior^19^ have been reported.

Hybrid quantum systems combining both cavity quantum electrodynamics and cavity optomechanics have been theoretically proposed^20,21^, with predictions of cooling at the single-polariton level, peculiar quantum statistics, and coupling to mechanical modes of both dispersive and dissipative nature. Cavity optomechanics with a polariton BEC opens intriguing perspectives, particularly in view of the potential access to an optomechanical strong-coupling regime, and the possibility of using vibrations to actuate on such a macroscopic quantum fluid. Indeed, the strength of the optomechanical interaction in conventional photon cavity optomechanics is quantified by the optomechanical cooperativity, $C=\frac{4{g}_{{\rm{0}}}^{{\rm{2}}}{n}_{{\rm{cav}}}}{\kappa {\Gamma }_{{\rm{m}}}}$. Here, *g*_0_ is the single-photon optomechanical coupling factor, *n*_cav_ is the number of photons in the cavity, and *κ* and Γ_m_ are the photon and mechanical decay rates, respectively^2^. For a polariton BEC one expects strongly enhanced values of the cooperativity *C* due to the very large coherent population of the condensate, coherence times that can be two orders of magnitude larger than the photon lifetime in the cavity, and optomechanical coupling interactions mediated by the excitons that can be resonantly enhanced^22^. Hybrid optomechanical systems based on BEC have, to the best of our knowledge, so far only been demonstrated with cold atoms in cavities coupled to low-frequency kHz vibrational degrees of freedom^23^.

It is the purpose of this paper to investigate the rich physics emerging from the coupling of a polariton BEC in a semiconductor microcavity with super-high-frequency vibrations confined in the same resonator^24,25^. The studied system contains GaAs/AlAs quantum wells (as two-level artificial atoms) coupled to cavity-confined optical and vibrational modes. Planar semiconductor microcavities are microstructured to laterally confine the polaritons, and the phonons, in stripes and in trap arrays. Optomechanically induced amplification (OMIA) experiments are presented to evidence the efficient coupling between the resulting exciton–polariton condensates to 20 GHz breathing-like vibrations confined in the same cavity. High-resolution spatially resolved low-temperature photoluminescence (PL) experiments evidence the emergence of mechanical self-oscillation, when polariton traps neighbor to the pumped one are red-detuned by even multiples of the confined phonon frequency. This observation highlights the relevance of high-order resonant optomechanical coupling in these devices. A theoretical model of resonant polariton-driven quadratic optomechanical coupling is introduced to describe the experimental observations. Conclusions are drawn showing that the findings contribute to developing new directions in the optomechanics and cavity-polariton fields, with applications in the technologically relevant extremely high-frequency range (30–300 GHz).

## Results

### The polariton optomechanical device

The studied system consists of cavity polaritons in μm-sized traps created by microstructuring the spacer of an (Al,Ga)As microcavity^26^. The lateral spacer modulation creates confinement potentials in 2D (wires and stripes), 3D (dots), as well as dot arrays consisting of non-etched areas surrounded by etched barriers (see a detailed description of the structure in the Supplementary Note 1). These polariton traps were studied by low-temperature PL in ref. ^26^. The sample is in the strong coupling regime both in the etched and non-etched regions, leading to microcavity polaritons in these two regions with different energies and photon/exciton content. BECs can be induced in the traps both with nonresonant and resonant excitation. As previously reported for similar planar^24^ and pillar^25^ microcavities, these structures also confine breathing-like longitudinal vibrations polarized along the growth direction, *z*, with a fundamental frequency around ${\nu }_{{\rm{m}}}^{{\rm{0}}} \sim 20$ GHz and overtones at ${\nu }_{{\rm{m}}}^{n}=(1+2n){\nu }_{{\rm{m}}}^{{\rm{0}}}$. We will concentrate here on experiments performed on a 40-μm-wide stripe (i.e., with weak lateral confinement), as well as on a square array of coupled square traps of 1.6 μm lateral size separated by 3.2-μm-wide etched regions.

### Optomechanically induced amplification

**Fig. 1:**
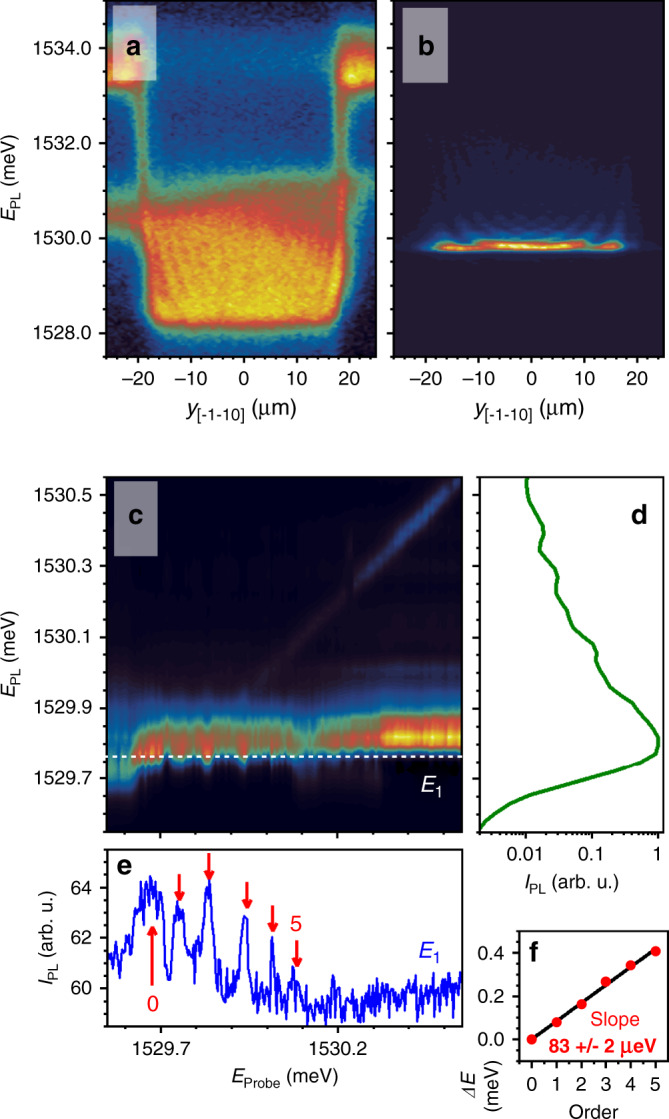
Bose–Einstein condensate optomechanical induced amplification. **a**, **b** Photoluminescence (PL) color maps of exciton–polaritons confined in a 40-μm-wide stripe. The two spatial images were recorded for nonresonant excitation powers well below (**a**, *P*_Pump_ = 10^−4^ *P*_Th_) and above (**b**, *P*_Pump_ = 2.3 *P*_Th_) the condensation threshold power *P*_Th_. **c** Color map of the spectral PL dependence on the probe laser energy. **d** The integrated PL intensity. **e** The emission amplitude of the fundamental BEC state as a function of the probe laser energy. The arrows highlight the peaks corresponding to the OMIA processes. Their energies as a function of the peak order are displayed in **f**.

Figure 1a, b shows the spectrally and spatially resolved PL image of the 40-μm-wide polariton stripe recorded at 5 K. The photonic mode in this microstructure is slightly negatively detuned with respect to the QW heavy-hole exciton state. The PL was nonresonantly excited using a Ti-Sapphire cw laser (1.631 eV) with pump powers *P*_Pump_ below, and above the condensation threshold power, *P*_Th_. At low powers (Fig. 1a, *P*_Pump_ = 10^−4^ *P*_Th_), the closely packed laterally confined levels in the stripe can be identified. Above threshold (Fig. 1b, *P*_Pump_ = 2.3 *P*_Th_), the polariton BEC is evidenced by the narrowing and concentration of the emission at the bottom of the trap, as well as by the blueshift of the states induced by polariton–polariton interactions mediated by their excitonic component, as well as polariton interactions with the excitonic reservoir^17^.

Figure 1c–f summarize the results of a two-laser OMIA experiment^27^, performed on this 40*-*μm-wide stripe. The first (pump) laser creates a BEC in the stripe. The second weak (probe) laser was scanned from higher to lower energies and through the condensate energy (details are provided in the Supplementary Note 2). The color map in Fig. 1c shows the PL intensity as a function of the probe laser energy. Figure 1d displays the integrated PL intensity, showing the emission of the closely spaced polariton states of the stripe. OMIA is evidenced by the variation of the PL intensity at the peak of the BEC emission as a function of the probe laser energy, which is also displayed in Fig. 1e. Notably, a series of peaks separated by the energy of the fundamental breathing-like cavity-confined vibrational mode (~20 GHz ~ 83 μeV) appears at the high-energy side of the BEC peak. A fit of the OMIA peak detunings, showing a clear lineal dependence, is presented in Fig. 1f. OMIA with the final state corresponding to the polariton fundamental energy is similar to a stimulated Raman process. The spectrum in Fig. 1e shows that the first and higher-order Stokes replicas (creation of one or several phonons) are amplified, but the anti-Stokes ones (annihilation of a phonon) are not. This asymmetry is attributed to the double optical resonance condition, which is satisfied for the Stokes processes, but not for the anti-Stokes ones^28^. In fact, the probe beam can only resonantly couple to modes with energy higher than that of the BEC. On the contrary, no modes are available at energies below the BEC, thus strongly suppressing this channel. The observation of up to five replicas of the 20 GHz phonon evidences a strikingly efficient optomechanical interaction.

### Mechanical self-oscillation

**Fig. 2:**
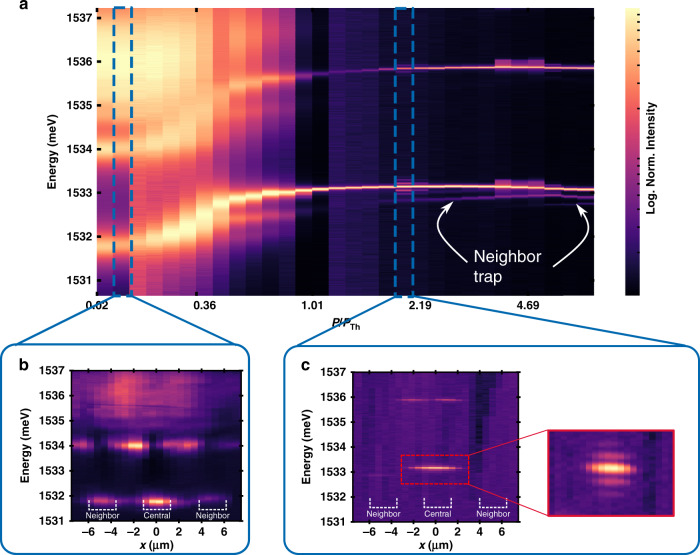
Bose–Einstein condensation in a polariton trap array. **a** Photoluminescence color map of exciton–polaritons confined in a square array of 1.6*-*μm-wide square traps as a function of the nonresonant excitation power (given in terms of the threshold power *P*_Th_ ~ 19 mW). For clarity each spectrum has been normalized to its maximum amplitude, and a logarithmic amplitude scale is used. The 3-μm-wide excitation spot was focused on a trap site: emission from both the pumped trap and from its redshifted neighbor traps can be clearly observed. The lower panels show the spatially resolved PL images for excitation below (**b**) and above (**c**) the BEC threshold. The emission from the neighboring traps can be clearly identified at *x* ~ ±4.8 μm in **b**. The inset in **c** shows the well-resolved vibrational sidebands.

To exploit such an efficient polariton–vibrational coupling for polariton-driven coherent phonon emission, we propose a double resonant scheme^5^, involving two confined states at neighboring sites of a square array of 1.6-μm-wide traps with a pitch of 4.8 μm. The color map in Fig. 2a displays the pump power dependence of the PL induced by a pumping spot focused onto a trap array. Spatially resolved PL images below and above threshold power are also presented (cf. Fig. 2b, c). While the ~3-μm-wide laser spot mainly addresses a single trap, neighboring traps can be excited through the tails of the laser Gaussian spot, as well as via polariton tunneling from the central trap and lateral propagation of the excitons in the reservoir. The fundamental and first excited states of the pumped trap, as well as weaker contributions from neighbor traps, can be identified in the color map and spatial images in Fig. 2. With increasing pump power the polariton modes blueshift. Above a threshold value, the intensity of the fundamental mode increases nonlinearly evidencing polariton BEC. Concomitantly with the nonlinear amplitudes increase, the emission linewidth narrows strongly. The measured linewidth is limited by the resolution of the triple-additive spectrometer. Measurements using a custom-made tandem Fabry–Perot–triple-additive spectrometer^29^, allow to access the true linewidth. The longest coherence time, observed at ~37 mW of pump power, is ~530 ps (linewidth ~8 μeV), two orders of magnitude larger than the polariton lifetime measured at low powers (~6–10 ps). Note that the neighbor traps also blueshift with increasing power, though with a weaker slope, thus attaining a power dependent redshift with respect to the pumped trap. This feature will become of critical relevance in what is discussed next.

The spatial image in Fig. 2c also shows that at some pump powers the BEC develops well-resolved equally separated sidebands, both on the high- and low-energy sides of the main peak. Such kind of sidebands are a signature of a coherent modulation of the BEC emission^30^, as previously observed for example for narrow emitters (semiconductor quantum dots^31^ and diamond NV centers^32^) externally driven by surface acoustic waves. The condition for the observation of these well-resolved sidebands is that the lifetime of the emitter exceeds the period of the modulation. It is particularly noteworthy that there is no external harmonic driving in the experiments displayed in Fig. 2, the only external source being the cw laser used for the nonresonant excitation of the semiconductor QWs.

**Fig. 3:**
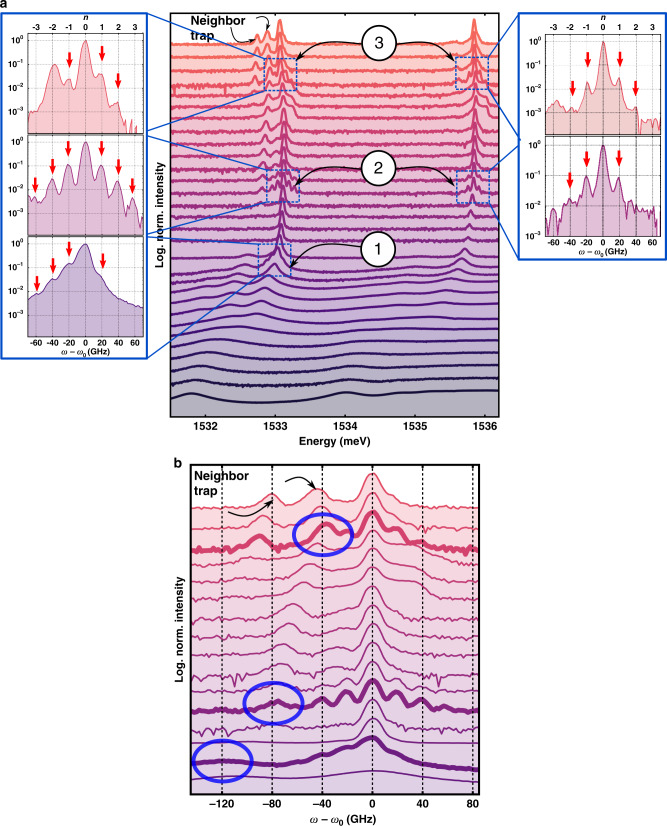
Regenerative mechanical self-oscillation induced by a BEC. **a** PL spectra for varying cw nonresonant excitation powers, for a 1.6 μm polariton square trap array. All spectra have been normalized to the maximum intensity. The numbered arrows highlight: (1) low-energy vibrational sidebands spaced by ${\nu }_{{\rm{m}}}^{{\rm{0}}}=20\,$ GHz ~ 83 μeV; (2) clearly resolved sidebands at both sides of the fundamental and first excited polariton BEC states; and (3) reappearance of the sidebands at a high excitation power. The left and right insets show details of the fundamental BEC and first excited polariton state, respectively. **b** PL spectra shifted in energy and shown relative to the fundamental BEC state, indicating PL contributions from neighbor traps. The spectra with pronounced sidebands (corresponding to the regions 1–3 in **a**) are highlighted with thicker traces. For these spectra, the energy of a neighbor trap (circular labels) is redshifted by an even multiple of the confined mechanical mode ${\nu }_{{\rm{m}}}^{{\rm{0}}} \sim 20$ GHz.

 Figure 3a shows the high-resolution spectra (in log intensity scale) for the full scan of measured powers, corresponding to the polariton trap-array PL shown in Fig. 2a. Again the blueshift and narrowing of the main BEC peak on increasing power can be clearly observed, together with the appearance of the redshifted PL from neighbor traps. The arrows with labels 1–3 in Fig. 3a and the side insets highlight excitation regions leading to: (1) intense and equally spaced low-energy sidebands, which are reminiscent of phonon assisted PL; (2) well-resolved sidebands on both sides of the polariton BEC at the fundamental and the first excited confined states; and (3) reapperance of the sidebands for both the fundamental and first excited polariton states at high powers.

**Fig. 4:**
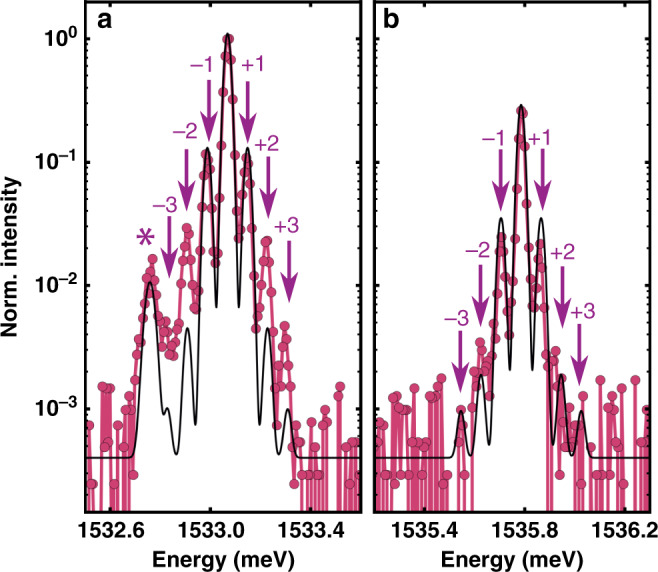
BEC optomechanics: self-oscillation and mechanical induced BEC sidebands. PL spectra of the fundamental (**a**) and first excited (**b**) polariton BEC mode with mechanically induced sidebands (red connected symbols). The solid thin black line is a fit to Eq. (1) yielding *χ* ~ 0.65 (see text for details). The asterisk indicates a peak due to PL from a neighbor trap, which was added ad hoc in the model.

The observed sidebands correspond precisely to equally spaced secondary peaks separated by the energy of the fundamental cavity-confined breathing mode (*ν*_0_ ~ 20 GHz ~ 83 μeV*)*. Under a coherent harmonic vibrational driving, the polariton BEC PL spectrum is expected to be proportional to *P*[*ω*] according to^31^:

1 $$P[\omega ]=\mathop{\sum }\limits_{{{n}}=-\infty }^{\infty }\frac{{J}_{{{n}}}^{{\rm{2}}}(\chi )}{{[\omega -({\omega }_{{\rm{BEC}}}-n{\omega }_{{\rm{d}}})]}^{2}+{\gamma }^{{\rm{2}}}},$$

that is, a sum of Lorentzians with linewidths 2*γ*, weighted by squared Bessel functions ${J}_{{{n}}}^{{\rm{2}}}(\chi )$. *χ* is a dimensionless parameter expressing the frequency shift on the BEC (*Δω*_BEC_) induced by the harmonic driving, stated in units of the driving frequency *ω*_d_ (*χ* = *Δω*_BEC_/*ω*_d_). The Lorentzians have maxima at frequencies *ω* = *ω*_BEC_ − *nω*_d_, where *n* is an integer. An example, Fig. 4a, b shows fits of Eq. (1) to the curves corresponding to the fundamental and excited BEC states, respectively, marked as 2 in Fig. 3a. The fits reproduce very well the measured spectral shape and yields *χ* = 0.65, and consequently *ΔE*_BEC_ ~ 55 μeV. From a calculation of the polariton energy dependence on strain (deformation potential (DP)) and cavity thickness (interface displacement) and using the value obtained for *ΔE*_BEC_, we estimate the amplitude of the coherent confined phonon field, and from there the average number of phonons (〈*N*〉) associated to the regenerative self-oscillation induced by the BEC (see the Supplementary Note 5 for details). We obtain a phonon number 〈*N*〉 ~ 2 × 10^5^, which far exceeds the thermal occupation of this mode at 5 K of 〈*N*〉_Thermal_ ~ 5, thus evidencing a very efficient polariton-to-phonon conversion. This phonon number corresponds to a maximum breathing of the cavity of ~660 pm, implying a strain of ~0.1%.

Coherent self-oscillation in cavity optomechanics is attained either when the external cw laser excitation is blueshifted with respect to the cavity mode in a single-cavity system or, in a coupled cavity system, when the higher cavity mode is excited^27^. The polariton BEC as an internal coherent source cannot thus, by itself, induce self-oscillation, because both Stokes and anti-Stokes processes would be equally probable. In our experiments, this symmetry is broken through the coupling of the BEC to the redshifted neighbor traps. Figure 3b shows details of the spectra from Fig. 3a, presented as a function of the detuning with respect to the BEC emission. The clearly resolved sidebands appear precisely when one of the neighbor traps is red-detuned by integer numbers of ${\nu }_{{\rm{m}}}^{{\rm{0}}} \sim 20$ GHz. The experimental observations can then be understood as follows: (i) the optomechanical interaction pumped by a confined BEC coupled to a precisely red-detuned neighbor trap (equivalent to a polariton population inversion) induces self-oscillations at the harmonics of the fundamental vibrational frequency ${\nu }_{{\rm{m}}}^{{\rm{0}}}=20\,$ GHz, and (ii) these coherent oscillation back-acts by modulating the BEC, thus leading to the observed sidebands. Furthermore, a closer look at Fig. 3b shows that the intensity of the phonon sideband increases when the neighbor trap is red-detuned by even numbers of ${\nu }_{{\rm{m}}}^{{\rm{0}}} \sim 20$ GHz: $\delta \nu =-2{\nu }_{{\rm{m}}}^{{\rm{0}}} \sim -40$ GHz, $\delta \nu =-4{\nu }_{{\rm{m}}}^{{\rm{0}}} \sim -80$ GHz, and $\delta \nu =-6{\nu }_{{\rm{m}}}^{{\rm{0}}} \sim -120$ GHz. This observation points to the relevance of both linear and quadratic terms in the optomechanical interaction^30,33^, as previously described, e.g., for the membrane in a cavity configuration^34,35^, a geometry to which the QWs in a cavity we are describing here can be mapped. Note that the transition to the self-oscillation regime could be controlled either by tuning the neighbors’ adequate energy level by the proper design of the system, so that their detuning is resonant with the mechanical frequency at a certain power, or by including an additional nonresonant pump laser to independently fine-tune the neighbors fundamental modes energy.

### Optomechanical model with linear and quadratic coupling

Polaritons exert force by radiation pressure (RP, i.e., through their photonic fraction) and by electrostriction (via the DP mediated by the excitonic component). The optomechanical coupling factor can be expressed as ${g}_{{\rm{0}}}={S}_{{\rm{x}}}\ {g}_{{\rm{0}}}^{{\rm{DP}}}+{S}_{{\rm{c}}}\ {g}_{{\rm{0}}}^{{\rm{RP}}}$, where *S*_x_(*S*_c_) denotes the exciton (photon) fraction of the polariton mode, and ${g}_{{\rm{0}}}^{{\rm{DP}}}({g}_{{\rm{0}}}^{{\rm{RP}}})$ the electrostrictive (RP) contribution to the optomechanical coupling factor (see the Supplementary Note 7). The excitons in the QWs can lead to a resonantly enhanced optical force^22^. In the studied sample, the QWs are positioned at the antinodes of the optical field in the unstructured regions to optimize the polariton strong coupling. The phonon strain vanishes at these positions, so that the DP coupling is not expected to contribute significantly except in the barriers between traps, where the asymmetric etching results in a departure from this cancellation. Evaluation of these forces shows that RP couples mainly to the fundamental 20 GHz mode, while electrostriction does with the overtone mode at ~60 GHz (see the Supplementary Note 6). We will thus consider in the following only the RP coupling leading to BEC modulation at harmonics of 20 GHz.

The theoretical model for the resonant linear coupling between two polariton modes presented in the Supplementary Note 7 yields an optomechanically modified phonon lifetime Γ_eff_ = Γ_m_(1 − *C*). The cooperativity *C* is formally equivalent to the conventional one presented in the introduction, but replacing *n*_cav_ by the number of polaritons in the pumped trap (*N*_1_), and interpreting *κ* as the decoherence rate of the polaritons in the traps. The optical excitation threshold for self-oscillations (i.e., corresponding to *C* > 1 (refs. ^2,27^)) is ${P}_{{\rm{Th}}}^{{\rm{so}}}=\frac{1}{\eta \times 1{0}^{7}}\frac{\kappa {\Gamma }_{{\rm{M}}}}{4{({g}_{{\rm{0}}}^{{\rm{RP}}})}^{2}} \sim \frac{0.7}{\eta }$ [mW] under double resonance conditions (see the Supplementary Note 7). *η* is the fraction of nonresonantly excited electron–hole pairs in the exciton reservoir that condense into the BEC. Assuming *η* = 40% (ref. ^36^), and a value of ${g}_{{\rm{0}}}^{{\rm{RP}}}$ previously reported for a pillar DBR microcavity^37^, this estimation yields ${P}_{{\rm{Th}}}^{{\rm{so}}} \sim 0.4$ mW. The hybrid polariton BEC optomechanical system already fulfills this conditions for self-oscillations at the BEC condensation threshold (*P*_Th_ ~ 19 mW for the 1.6 μm traps). We note however that ${g}_{{\rm{0}}}^{{\rm{RP}}}$ was calculated for two modes of an isolated pillar. For the studied trap array, the initial and final polariton states belong to spatially separated traps. The penetration of the polariton ground states in the barriers, obtained from realistic calculations based on the low-power known trap potential^26^, amounts to ~1 μm for low excitation densities. The separation between traps in the studied array is 3.2 μm and thus the overlap integral based on these trap ground state wavefunctions should be small. The spatial images at the powers where self-oscillation is observed evidence a large transfer of polaritons between traps (see the Supplementary Note 8 and the Supplementary Fig. 7). We speculate that repulsive interactions may modify the polariton distribution within the traps^38^, as well as blueshift the polariton levels, thus enhancing the tunnel coupling between neighboring traps.

While the previously described linear coupling process can explain phonon lasing, the experiments also suggest that a quadratic coupling is relevant. Namely, the self-oscillations become strongly enhanced at detunings corresponding to even harmonics of ${\nu }_{{\rm{m}}}^{{\rm{0}}} \sim 20$ GHz. Under these conditions a BEC also forms at the neighbor trap, thus implying stimulation into the final state in this trap. We have evaluated the conditions for self-oscillation for the quadratic resonant coupling between two polariton states separated by $2{\nu }_{{\rm{m}}}^{{\rm{0}}}$ (see the Supplementary Note 7), assuming a polariton–phonon quadratic coupling strength *G*_2_. The effective phonon linewidth is in this case given by

2 $${\Gamma }_{{\rm{eff}}}=2\ {\Sigma }_{0}\left(| {\Sigma }_{0}{| }^{2}-| {\Sigma }_{1}{| }^{2}\right)/\left(| {\Sigma }_{0}{| }^{2}+| {\Sigma }_{1}{| }^{2}\right)$$

with

3 $${\Sigma }_{0}\equiv \frac{{\Gamma }_{{\mathrm{m}}}}{2}\left(1-\frac{16{G}_{2}^{2}({N}_{1}-{N}_{2}){N}_{{\rm{b}}}}{\tilde{\kappa }{\Gamma }_{{\mathrm{m}}}}\right),$$

4 $$\left|{\Sigma }_{1}\right|\equiv 2{G}_{2}\sqrt{{N}_{1}{N}_{2}}\left|1-\frac{16{G}_{2}^{2}{N}_{{\rm{b}}}^{2}}{\tilde{\kappa }\kappa }\right|,$$

where $\tilde{\kappa }\equiv \kappa +\frac{4{G}_{2}^{2}{N}_{{\rm{b}}}^{2}}{\kappa }$, describes the phonon-induced broadening of the polariton modes. According to Eq. (2), self-oscillations start when either Σ_0_ or the factor ${\left|{\Sigma }_{0}\right|}^{2}-{\left|{\Sigma }_{1}\right|}^{2}$ reduces to zero. The quadratic coupling self-oscillation threshold depends not only on the external pumping rate (through *N*_1_), but also on the steady-state number of phonons (*N*_b_) and final state polaritons (*N*_2_). From the intensity of the neighbor trap emission under self-oscillation in Fig. 3, we estimate that a quadratic coupling constant *G*_2_ = 2*π* × 1 Hz suffices to induce a self-oscillation threshold power of 10 mW. This *G*_2_ is more than two orders of magnitude smaller than the quadratic coupling constants previously reported for other optomechanical devices^33^, thus providing plausibility to the proposed mechanism.

## Discussion

We have demonstrated coherent phonon generation and self-modulation at ultra-high vibrational frequencies using a two-mode polariton BEC. In contrast to other demonstrations of laser-induced regenerative self-oscillations of a phonon mode^2,4,5^, the self-oscillations in our scheme are driven by an internally emitting (population inverted) polariton condensate, a source that can be electrically pumped^18^. The coherently generated phonons are efficiently emitted into the supporting substrate^39^, thus providing a new platform for an electrically driven parametric phonon laser. The cavity quantum electrodynamics features of the system result in a strong energy-conserving coupling between photons and excitons, while the optomechanics term couples off-resonant mechanical and photonic modes of widely different frequencies (GHz and hundreds of THz, respectively). Thus, the demonstrated polariton platform can be at the base of novel technologies for frequency conversion between light and mechanical (or microwave) signals in the 20 GHz range^10,11,13,40^. Similarly, our findings demonstrate that mechanical vibrations can coherently actuate on a macroscopic quantum fluid (the BEC) at frequencies exceeding its decoherence rate. The strength of the coupling can be enhanced by several orders of magnitude exploiting the resonant character of the photoelastic coupling mediated by excitons in QWs^22^. Finally, the present optomechanics platform enables coupling to cavity mechanical modes of hundreds of GHz^41^, thus providing access to operation and signal transduction at the so-called extremely high-frequency range.

## Methods

### Experimental details

For the two-laser OMIA type experiments in the 40-μm-wide polariton stripe at 5 K, a cw Spectra Physics Ti-Sapphire Matisse laser was used for the nonresonant excitation at 1.631 eV. A second weaker Toptica semiconductor stabilized laser, incident with a finite angle, was tuned around the energy of the BEC, and light was collected along the normal to the sample. The high-resolution spectroscopic experiments in the trap array were performed at 5 K with cw nonresonant excitation (1.631 eV), with a microluminescence setup based on a ×20 microscope objective (NA = 0.3, spot size ~3 μm) and a triple-additive spectrometer (resolution ~0.15 cm^−1^ ~20 μeV).

### Reporting summary

Further information on research design is available in the Nature Research Reporting Summary linked to this article.

## Supplementary information

Supplementary Information

Reporting Summary

## Data Availability

The Source data that support the findings of this study are available from the corresponding author upon reasonable request. All these data are directly shown in the corresponding figures without further processing.
