# Supplementary material for: Polariton-driven phonon laser
Source: Nat Commun. 2020 Sep 11;11:4552. doi: 10.1038/s41467-020-18358-z (PMC7486378; doi:10.1038/s41467-020-18358-z)
Supplement: Supplementary file 1 — Supplementary Information [file 41467_2020_18358_MOESM1_ESM.pdf]

# Supplementary Information: Polariton-Driven Phonon Laser

D. L. Chafatinos,[1] A. Kuznetsov,[2] S. Anguiano,[1] A. E. Bruchhausen,[1]
A. A. Reynoso,[1] K. Biermann,[2] P. V. Santos,[2] and A. Fainstein[1,*]

[1]*Centro Atómico Bariloche & Instituto Balseiro (CNEA) and CONICET, and Universidad Nacional de Cuyo (UNCuyo), Av. E. Bustillo 9500, R8402AGP S.C. de Bariloche, R.N., Argentina.*

[2]*Paul-Drude-Institut für Festkörperelektronik, Leibniz-Institut im Forschungsverbund Berlin e.V., Hausvogteiplatz 5-7, 10117 Berlin, Germany.*

## SUPPLEMENTARY NOTE 1: STUDIED STRUCTURE.

The studied device consists of polaritons in arrays of $\mu$m-sized intracavity traps created by patterning an (Al,Ga)As microcavity in-between growth steps by molecular beam epitaxy (see a scheme in Supplementary Fig. 1).[1] First a 4.43-$\mu$m thick lower distributed Bragg reflector (DBR) consisting of 36 $\lambda/4$ ($\lambda$ is the optical wavelength) pairs of $Al_{0.15}Ga_{0.85}As/Al_xGa_{1-x}As$ with the Al composition $x$ continuously reducing from 0.80 in the first stack to 0.45 in the last stack. The first 120 nm of the $Al_{0.30}Ga_{0.70}As$ microcavity spacer were then deposited including six 15-nm-thick

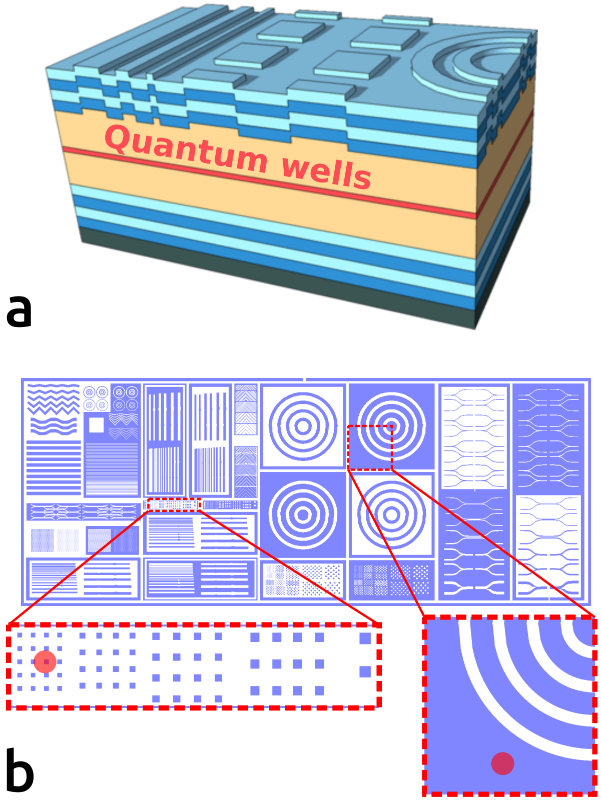


**Supplementary Figure 1**. **Studied microstructured planar microcavity.** Panel (a) presents a sketch of the (Al,Ga)As microcavity with the structured spacer enclosing GaAs QWs grown on a GaAs(001) substrate. The thickness of the spacer between the distributed Bragg reflectors (DBRs) was varied by combining etching and overgrowth by molecular beam epitaxy, resulting in regions with different polariton energies. Panel (b) shows a detail of the two microstructures studied in this work, an extended region similar to a stripe of $\sim$ 40$\mu$m thickness, and an array of 1.6$\mu$m square traps separated by 2$\mu$m edged barrier regions. The red circles mark approximately the regions probed in the reported experiments.

GaAs quantum wells (QWs) placed at the antinode positions of the microcavity optical mode. The structure was subsequently capped by a 170-nm-wide $Al_{0.15}Ga_{0.85}As$ layer spacer.

The sample was then taken out of the molecular beam epitaxy (MBE) chamber and then patterned by means of photolithography and wet chemical etching. The latter creates mesas with a nominal height of 12 nm of different shapes in the exposed spacer layer (see Supplementary Fig. 1), thus inducing a lateral modulation of the cavity thickness and, therefore, of the cavity energy in the final structure. The etching depth results in a blueshift of the optical cavity mode in the etched areas by 9 meV (4.5 nm) with respect to the non-etched regions. The upper surface of the etched layer corresponds to a node of the optical cavity mode of the whole structure. In this way, potential impact of roughness or impurities introduced by the ex situ patterning on optical properties of the structure was minimized. Furthermore, the shallow patterned layer is located more than 140 nm above the QWs, so that they remain unaffected by the processing. The sample was then reinserted into the MBE system, cleaned by exposure to atomic hydrogen, and overgrown with a $\lambda/4$ $Al_{0.15}Ga_{0.85}As$ layer, followed by the upper DBR. The latter consists of 20 $\lambda/4$ pairs of $Al_{0.15}Ga_{0.85}As/Al_{0.75}Ga_{0.25}As$. The sample is in the strong coupling regime both in the etched and nonetched regions, leading to microcavity polaritons in these two regions with different energies and photon/exciton contents.[1] The lateral modulation was used to create 2D (stripes and wires) and 3D (dots) confinement in nonetched areas surrounded by etched barriers, as probed by low-temperature reflection and photoluminescence (PL) in Ref.[1]. Panel (b) in Supplementary Fig. 1 illustrates the two microstructured regions in the sample that were studied for the present investigation of polariton BEC optomechanics, a stripe of 40$\mu$m thickness (very weak lateral confinement), and an array of small coupled square traps of 1.6$\mu$m size, separated by 2$\mu$m etched regions.

## SUPPLEMENTARY NOTE 2: EXPERIMENTAL SET-UPS.

Two different set-ups were used, one for the measurement of the two-laser OMIA type of experiments in the 40$\mu$m thickness stripe, the other for the high-resolution optomechanical spectroscopy of the 1.6$\mu$m square trap array.

Supplementary Figure 2 describes the setup used for the two-laser OMIA type experiments in the 40$\mu$m polariton stripe. A *cw* Spectra Physics Ti-Sapphire Matisse laser is used for the non-resonant excitation at 760nm. A second weaker Toptica semiconductor stabilized laser, incident with a finite angle, is tuned around the energy of the Bose-Einstein condensate, and light is collected along the normal to the sample.

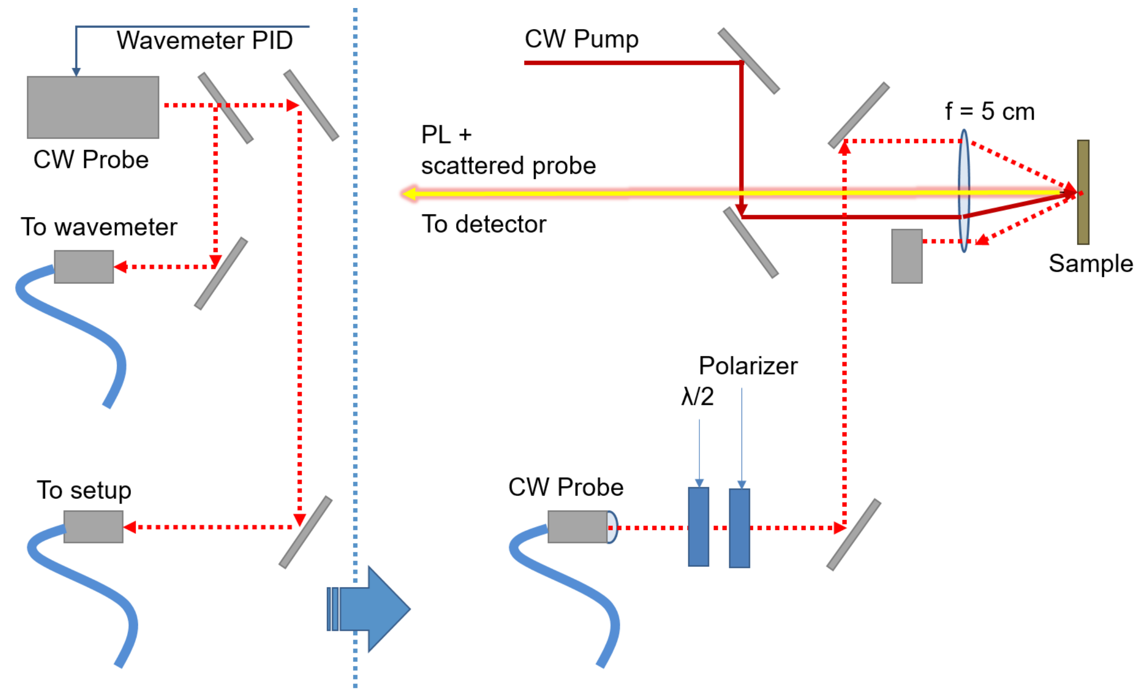


**Supplementary Figure 2**. **Two-laser OMIA setup.** Scheme of the experimental setup used for the two-laser OMIA type experiments in the 40$\mu$m polariton stripe.

The frequency of the cavity-confined breathing type of mechanical vibrations is large for the typical noise measurements used in lower frequency cavity optomechanics experiments, but rather small for standard optical spectroscopy methods. This imposes strong requirements for the spectral resolution and bandwidth of the vibrational spectroscopy used. To this aim we have used both a photoluminescence microscopy set-up coupled to a triple additive spectrometer (based on three stages of 64 cm each one, and three 1800 gr/mm holographic gratings), and a purposely developed Raman spectroscopy technique based on a tandem Fabry-Perot triple spectrometer multichannel set-up (see the scheme in Supplementary Fig. 3).[2] The latter system is composed of a single-pass Fabry-Perot (FP) interferometer coupled to the T64000 Jobin-Yvon triple spectrometer operated in additive configuration.[2] The light to be analyzed is collected from the sample by a lens, filtered through the FP, and then focused by a second lens into the entrance slit of the spectrometer. The FP contains two high-quality ($\lambda/200$) dielectric mirrors for the near infrared (99% peak reflectivity centered at 870 nm), which are kept parallel at a fixed distance by three high-quality ($\lambda/200$) cylindrical silica spacers. The mirrors are located in a sealed chamber connected to a pure Nitrogen gas distribution and vacuum system. As the resolution of the spectrometer is better than the free

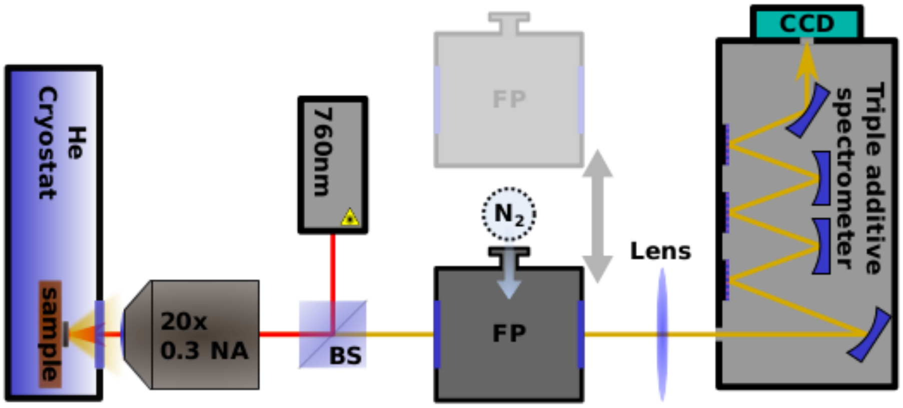


**Supplementary Figure 3**. **High resolution micro-photoluminescence setup.** Scheme of the experimental setup used for the high-resolution experiments in the array of coupled square traps of 1.6$\mu$m. It comprises a standard microscope photoluminescence configuration with a 20x NA=0.3 objective, a Spectra Physics Ti-Sapphire Matisse laser, and a cold-finger He cryostat, plus two high-resolution features: i) a triple additive T64000 Jobin-Yvon spectometer with 1800 gr/mm gratings, and ii) the possibility to include a home-made tunable Fabry-Perot interferometer in the collection path for sub-pixel resolution (see text for details).

spectral range (FSR) of the FP but not enough to resolve the width of its transmission peaks, the acquired spectrum consists of several broad resolution-limited peaks of which the relevant information is their integrated intensity. By repeating this procedure as a function of the gas pressure, we reconstruct the photoluminescence profile with a sub-pixel resolution improved by almost two orders of magnitude.[2] The triple spectrometer is equipped with a liquid-$N_2$ cooled charge-coupled device (CCD) multichannel detector which allows for the parallel acquisition of the spectra transmitted through a large set of FP resonances. The excitation is done using a near-infrared Ti:sapphire single-mode Spectra- Physics Matisse TS ring laser, the wavelength of which can be locked to an external confocal cavity with a precision better than $2 \times 10^{-6}$ cm$^{-1}$. With this set-up the resolution of the triple spectrometer was improved from $\sim 0.5$ cm$^{-1}$ $\sim 15$ GHz to $\sim 3 \times 10^{-3}$ cm$^{-1}$ $\sim 90$ MHz.

## SUPPLEMENTARY NOTE 3: POWER DEPENDENCE OF AMPLITUDE AND LINEWIDTH OF THE BEC EMISSION.

We present in this section some details of the intensity and linewidth of the BEC emission in the array of small coupled square 1.6$\mu$m traps described in Fig. 2 of the main text. The left panel in

Supplementary Fig. 4 shows the Q-factor associated to the BEC emission peak, as determined from the luminescence measurements at 5 K using both the high-resolution triple additive spectrometer (TAS, red triangles in the figure), and the ultra-high-resolution Fabry-Perot-Triple-Spectrometer tandem (TAS+FP, black solid circles). The TAS measurements saturate at $Q \sim 5\times10^4$ marking the resolution limit of the high-resolution setup ($\sim 20\mu$eV). The use of the ultra-high resolution allows to access the true-linewidth ($\sim 8\mu$eV), corresponding to $Q \sim 2 \times 10^5$ and a BEC coherence time $\tau_{coh} \sim 530$ ps. The right panel in Supplementary Fig. 4 presents the intensity of the BEC emission peak as a function of the pump power. The inset is displayed in logarithmic scale to emphasize the non-linear dependence and threshold behavior. The main panel present the same results in linear scale, with down red arrows highlighting dips that correlate with the regions in which $\nu_m^0 = 20$ GHz mechanical sidebands are evidenced in Fig.3 of the main text. The blue up-arrow corresponds to $-3\nu_m^0 = \nu_m^1 = -60$ GHz, where also a dramatic change of the BEC emission spectra is apparent in Fig.3 of the main text which, however, has not been yet completely understood. The dips in the BEC emission amplitude evidence a transfer of spectral weight from this mode to the neighbor traps and to the sideband resolved optomechanical secondary peaks.

## SUPPLEMENTARY NOTE 4: CALCULATION OF $g_0$.

For the calculation of the radiation pressure contribution to the optomechanical coupling factor $g_{om}^{RP}$, we followed the analysis proposed by Johnson *et al*,[3] implementing a finite element-method to obtain the electric and acoustic fields. We generalized the approach presented in Refs. [4] and [5] to compute the effects induced by the multiple interfaces at the DBR's boundaries,[6]

$$g_{om}^{RP} = \frac{\omega_c}{2} \sum_i \frac{\oint_{A_i} (\vec{u} \cdot \hat{n}_i)(\Delta\epsilon_i |\vec{E}_\parallel|^2 - \Delta(\epsilon_i^{-1})|\vec{D}_\perp|^2) dA_i}{\int \epsilon |\vec{E}|^2 d\vec{r}}, \tag{1}$$

where $\omega_c$ is the optical angular frequency at resonance, $\vec{u}$ the normalized displacement field, $\hat{n}_i$ the unitary normal-surface vector corresponding to the interface, $\Delta\epsilon_i = \epsilon_{i,left} - \epsilon_{i,right}$ the difference between the dielectric constants of the materials involved, $\Delta\epsilon_i^{-1} = \epsilon_{i,left}^{-1} - \epsilon_{i,right}^{-1}$, $\vec{E}_\parallel$ is the component of the electric-field parallel to the interface surface and $\vec{D}_\perp$ is the normal component of the displacement field $\vec{D} = \epsilon_0\epsilon_r\vec{E}$. The index $i$ runs over every distinct interface $A_i$.

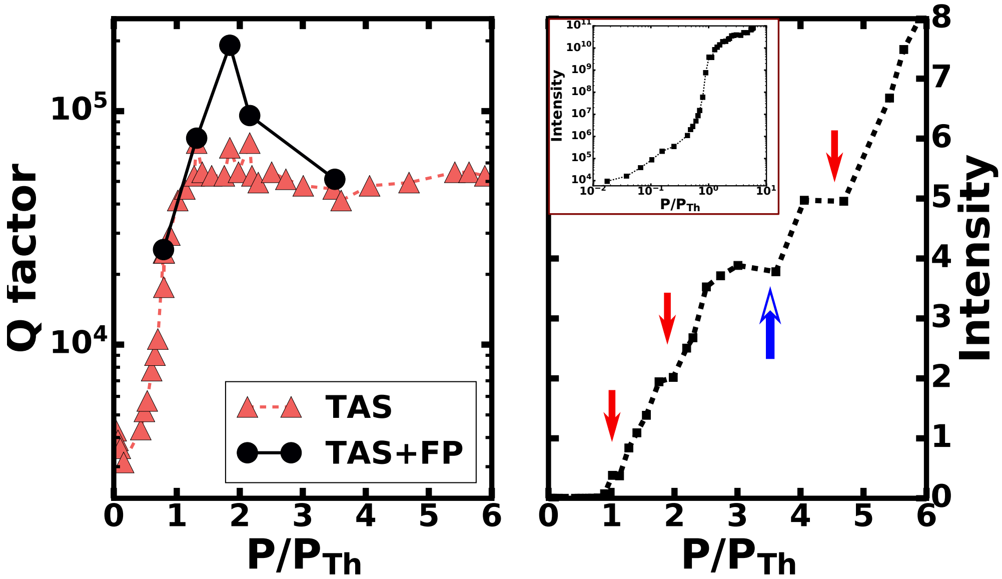


**Supplementary Figure 4. Intensity and Q-factor of the BEC emission of the coupled square 1.6$\mu$m array.** Left panel: Polariton Q-factor determined from luminescence measurements using the high-resolution triple additive spectrometer (TAS, red triangles), and the Fabry-Perot-Triple Additive Spectrometer tandem (TAS+FP, black circles). Above threshold the measurements with the TAS saturate at $Q \sim 5 \times 10^4$ due to experimental resolution. The true linewidth (and hence Q-factor) requires the use of the ultra-high-resolution tandem. Right panel: Intensity of the BEC emission as a function of the non-resonant pump power. The inset shows the curve in logarithmic scale to emphasize the non-linear behavior and the threshold power. Arrows highlight dips that correlate with the regions in which mechanical signatures appear in Fig.3 of the main text.

## SUPPLEMENTARY NOTE 5: DISPLACEMENT AND AVERAGE PHONON NUMBER ASSOCIATED TO THE REGENERATIVE SELF-OSCILLATION.

Using Eqn.(1) of the main text, as shown in Fig.4, the emitted spectra have been fitted yielding a dimensionless parameter $\chi = 0.65$. The driving angular frequency $\omega_d$ corresponds to the coherent mechanical cavity confined fundamental breathing mode ($\hbar\omega_d \simeq 80\mu eV$), which induces an energy shift of the optical mode $\Delta E_{BEC} = \hbar\Delta\omega_0 = \hbar\omega_d\ \chi \sim 55\mu eV$.

The presence of the confined acoustic mode, induces a shift of the interfaces of the structure, and this expansion/contraction modifies the interaction with the electromagnetic fields, i.e. with the cavity exciton-polatiton system. In order to estimate the average number of acoustic phonons $\langle N \rangle$ driving the system, together with the associated strain, we proceed the following way.

The electromagnetic fields are calculated assuming a planar structure, using a conventional transfer matrix formalism (TMF), including a complex dielectric susceptibility that accounts for

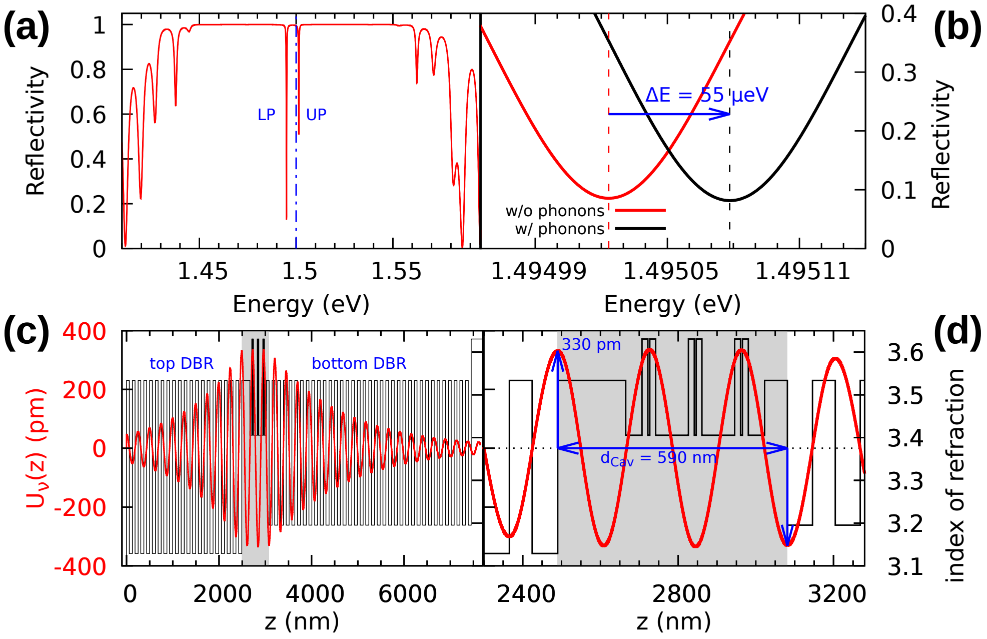


**Supplementary Figure 5**. **Evaluation of the phonon induced cavity energy shift.** Panel (a): Calculated optical reflectivity spectrum including an exciton state and a slightly negatively detuned cavity mode. The resulting coupled modes (lower and upper polatitons, LP and UP) are observed. Panel (b): Estimate LP mode's energy shift matching the experimentally estimated energy shift of $55\mu eV$, corresponding to a coherent self-oscillating vibrational mode with phonon average occupation number $\langle N \rangle \sim 2 \times 10^5$. Panel (c): Calculation of the cavity confined fundamental breathing mode at 20 GHz. Panel (d): Detail of the cavity spacer's region indicating the vibrational mode that induces a symmetric expansion-contraction of the spacer (vertical blue arrows). The magnitude of the displacement corresponds to $\langle N \rangle \sim 2 \times 10^5$ and leads to the energy shift displayed in panel (b).

the excitonic states of the embedded quantum wells. Based on the TMF, the optical reflectivity spectrum is calculated and depicted in Supplementary Fig. 5(a). Here, both resulting modes (lower and upper polatiton modes, LP and UP) are observed, and represent a situation of a slight negative cavity-exciton detuning.

The approach used is quasi-static, i.e. the optical frequencies are high enough compared to the frequencies of the phononic system to consider the vibrations as "frozen". The cavity mechanical acoustic vibrations are calculated using an elastic continuum one-dimensional model, also using conventional TMF, and considering the corresponding elastic boundary conditions and the mode's normalization. The calculation for the cavity confined fundamental breathing mode is shown in Supplementary Fig. 5(c). The acoustic modes displacement (in red) is superimposed to the profile of the structure's index of refraction. The region of the cavity is shaded in grey in between the

top/bottom DBRs. Here, $z = 0$ corresponds to the air-sample surface. Supplementary Figure 5(d), shows a detail of the cavity spacer's region of Supplementary Fig. 5(c). As observed, the vibrational mode induces a symmetric expansion-contraction of the spacer (vertical blue arrows).

The value of the acoustic displacement field $U(z)$ at the interfaces between the different materials indicate the modification of the un-perturbed structure. This modification of the structure given by the effective expansion/contraction of the layers is considered for calculating the perturbed optical reflectivity spectrum. In Supplementary Fig. 5(b) a zoom of the LP cavity mode is presented. As observed, the action of the acoustic strain (in its contraction state) is to shift the optical mode to higher energies. To estimate the polariton mode's energy shift $\Delta E_{BEC}$, we increased the number of phonons $\langle N \rangle$ populating this mechanical confined mode, to match the experimentally estimated energy shift of $55\mu eV$ [see Supplementary Fig. 5(b)]. The estimated number corresponds to $\langle N \rangle \sim 2 \times 10^5$, and the resulting confined mode is shown in Supplementary Fig. 5(d), where the phonon's amplitude at each cavity interface is $\sim 330$ pm. Given the fact that the nominal cavity width is $d_{cav} = 590$ nm, the associated strain is of the order of 0.1%.

Summing-up then, the proposed model provides the means to establish the magnitude of the self-oscillation, and thus of the number of cavity phonon modes present:

1. From the amplitude of the side-oscillations relative to the main peak, we extract $\chi$;

2. From $\chi$, we determine the magnitude of the energy modulation of the BEC emission;

3. From this latter value, and a relatively simple calculation of the perturbation induced on the polariton cavity modes by the breathing associated to the vibrational cavity mode, we determine the amplitude of the existent phonon field. For this purpose the polariton modes of the structure are derived from solving Maxwell's equations using the transfer matrix method for the electromagnetic fields, including all the DBR layers as well as the resonant QWs.[7] The vibrational acoustic modes of the structure, in turn, are obtained from solving the elastic continuum mechanical problem, also using the transfer matrix method, likewise accounting for all the structure's layers.[8,9] For both cases the real nominal structure of the device and standard material parameters are used.

4. Based on the above described solution of the mechanical excitations of the device, the amplitude of the displacement associated with a single phonon can be calculated. Thus, from the amplitude of the phonon field determined in the previous step, the number of coherent phonons present in the self-oscillation can be determined.

We stress that this derivation is parameter-free.

## SUPPLEMENTARY NOTE 6: RADIATION PRESSURE (RP) VS. ELECTROSTRICTION (ES) SPECTRA IN NON-ETCHED / ETCHED SAMPLE REGIONS.

We discuss in this section the amplitude of the different cavity vibrational modes depending on the mechanism of light-matter interaction, namely radiation pressure and exciton-mediated electrostriction. The calculations were performed on a model structure with layer thicknesses somewhat smaller than the precise microstructure reported in the paper. This explains the slightly larger magnitude of the calculated vibrational frequencies, when compared to the experiments reported in the paper. The models used are the same as described in Ref.[6].

In Supplementary Fig. 6(a) the profile of the index of refraction ($n$, dark-green lines) shows the detail of the cavity spacer (gray-shaded area) of the structure. The two regions of the sample are analyzed: The top panel corresponds to the non-etched region (nER), while the bottom panel to

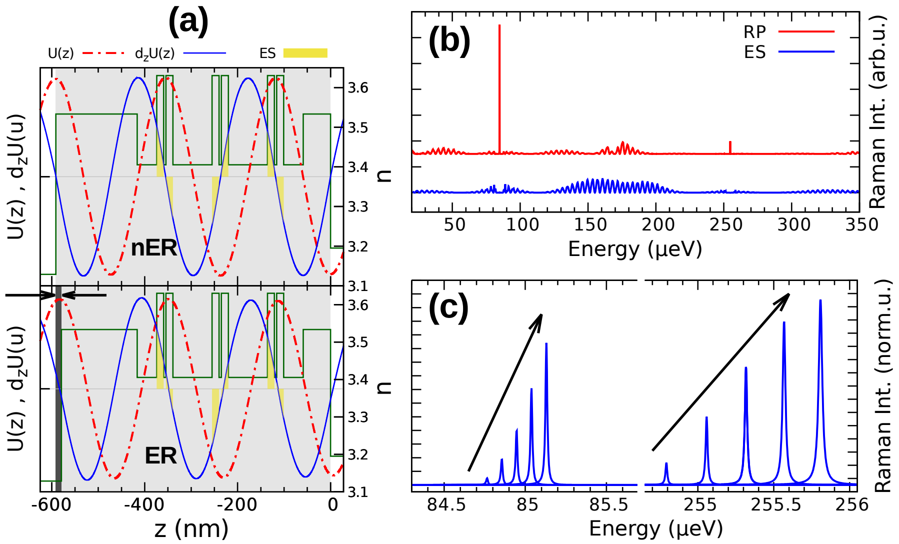


**Supplementary Figure 6. Vibrational spectra due to radiation pressure and electrostriction optical forces.** Panel (a): Acoustic displacement field $U(z)$ (dashed-dotted red curve) of the cavity confined mechanical modes, together with their associated strain $d_zU(z)$ (blue curves). The two regions of the sample are analyzed, non-etched (nER), and etched regions (ER). The profile of the index of refraction ($n$, dark-green lines) shows the detail of the cavity spacer (gray-shaded area) of the structure. Panel (b): Vibrational spectra of both radiation pressure (RP, red curve) and electrostriction (ES, blue curve) forces, corresponding to the non-etched regions. Panel (c): Evolution of the Raman polarizability due to electrostriction interaction when the cavity spacer is increasingly etched.

the etched region (ER), i.e. to the region surrounding the cavity polariton traps. Superimposed for both cases, the acoustic displacement field $U(z)$ (dashed-dotted red curve) of the cavity confined mechanical modes are shown, together with their associated strain $d_zU(z)$ (blue curves). The region that is edged out (bottom panel) is indicated by the horizontal black arrows. The $U(z)$ of the nER (top panel), is the same field displayed in Supplementary Fig. 5. One important point to notice here is the fact that for the nER the mechanical modes' antinodes fall precisely in between the quantum well (QW) pairs. As a consequence, the associated strain $d_zU(z)$ has a node there. As we will explain next, this fact has drastic consequences for one of the polariton-phonon interaction mechanisms, i.e. for the electrostriction (ES) process (via the deformation potential interaction). This interaction, is basically proportional to the integrated strain present at the QWs (region marked with yellow). As can be observed, due to the fields' symmetry with respect to the QWs, for the nER, the areas contributing positively are canceled out by those contributing negatively. While the radiation pressure process (RP) is only influenced by the effective expansion/contraction of the cavity, which is only very weakly affected by the etching, for electrostriction (ES) at the polariton traps (nER) the effect is practically zero. Interestingly, when the etching takes on, the symmetry breaks down and the strain for the QWs is modified yielding a non-zero effective contribution.

In Supplementary Fig. 6(b) we compare the contributions to the vibrational spectra of both radiation pressure (RP, red curve) and electrostriction (ES, blue curve) corresponding to the non-etched regions. These curves are obtained from the corresponding overlap integral between the initial and scattered electromagnetic field[6], which is proportional to the Raman polarizability. As can be seen, RP shows a very strong coupling to the confined modes at $\sim$22 GHz ($\sim 85\,\mu$eV) and at $\sim$ 66 GHz($\sim 255\,\mu$eV), while the contribution for ES is absent (no peaks can be observed at these frequencies).

To show the increasing coupling when passing from one region to the other, in Supplementary Fig. 6(c) we analyze the evolution of the Raman polarizability due to electrostriction interaction when the cavity spacer is progresively etched. It can be well seen that the coupling via electrostriction increases rapidly and significantly ($\sim \times 30$) when the spacer is reduced (following the black arrows), affecting the coupling to the fundamental $\nu_m^0$ and the second overtone $\nu_m^1$. The etching steps in Supplementary Fig. 6(c) are 2 nm, starting from the nominal non-etched structure (nER in Supplementary Fig. 6(a)) and towards the situation described in Supplementary Fig. 6(a) bottom panel (ER). Notice that in fact the vibrational mode at $\sim$66 GHz($\sim 240\,\mu$eV) actually couples more efficiently to the cavity polariton via ES than the fundamental mechanical mode.

## SUPPLEMENTARY NOTE 7: TWO-POLARITON MODE HAMILTONIAN COUPLED TO LINEAR AND QUADRATIC OPTOMECHANICAL INTERACTIONS.

The considered Hamiltonian

$$\hat{H}_0 = \hat{H}_1 + \hat{H}_2 + \hbar\omega_m \hat{b}_m^\dagger \hat{b}_m + \hat{H}_{\text{int}} \tag{2}$$

describes two isolated traps coupled to a phonon mechanical mode of frequency $\omega_m \equiv 2\pi\nu_m^0$ with bosonic annihilation (creation) operator $\hat{b}_m$ ($\hat{b}_m^\dagger$). The Hamiltonians of the traps are

$$\hat{H}_i = \hbar\omega_{e,i}\hat{e}_i^\dagger\hat{e}_i + \hbar\omega_{c,i}\hat{c}_i^\dagger\hat{c}_i + \frac{1}{2}\hbar\xi_i(\hat{c}_i^\dagger\hat{e}_i + \hat{e}_i^\dagger\hat{c}_i), \tag{3}$$

where $\hat{e}_i$ ($\hat{e}_i^\dagger$) and $\hat{c}_i$ ($\hat{c}_i^\dagger$) are the bosonic annihilation (creation) operators of the cavity exciton and cavity photon fields, respectively. In each trap, $\xi_i$ accounts for the exciton-photon coupling, and $\omega_{e,i}$ ($\omega_{c,i}$) accounts for the cavity exciton (photon) frequency.

The interaction between the traps and the phonon mode includes a radiation pressure and an electrostriction (via the deformation potential, DP) contribution through the coupling strengths $g_0^{RP}$ and $g_0^{DP}$, respectively. These parameters account for the mechanisms described in Supplementary Notes (4)-(6) above. We write the interaction as

$$\hat{H}_{\text{int}} = -\hbar g_0^{RP}\hat{c}_2^\dagger\hat{c}_1\hat{b}_m^\dagger - \hbar g_0^{DP}\hat{e}_2^\dagger\hat{e}_1\hat{b}_m^\dagger + h.c. \tag{4}$$

where we assumed that the first (1) polariton BEC mode is blue-shifted with respect to second trap (2), and followed our measurements insight that the Stokes process is due to mixing of two neighbor trap modes in the optomechanic (OM) coupling.

Using the polariton basis each trap Hamiltonian becomes diagonal,

$$\hat{H}_i = \hbar\Omega_{i,+}\hat{p}_{i,+}^\dagger\hat{p}_{i,+} + \hbar\Omega_{i,-}\hat{p}_{i,-}^\dagger\hat{p}_{i,-}\ , \tag{5}$$

where $2\Omega_{i,\pm} = \omega_{c,i} + \omega_{e,i} \pm \sqrt{(\omega_{c,i} - \omega_{e,i})^2 + \xi_i^2}$ and the polariton annihilation operators are, $\hat{p}_{i,+} = \cos\theta_i\hat{c}_i + \sin\theta_i\hat{e}_i$ and $\hat{p}_{i,-} = \cos\theta_i\hat{e}_i - \sin\theta_i\hat{c}_i$ with $2\theta_i = \arctan(\xi_i/(\omega_{c,i} - \omega_{e,i}))$. For $\omega_{c,i} = \omega_{e,i}$ one has $\theta_i = \pi/4$ i.e., the polaritons' excitonic and photonic components are equal. On the other hand for $\omega_{c,i} \ll \omega_{e,i}$ one has $\theta_i \approx \pi/2$ and the low polariton (LP) solution becomes photonic: $\hat{p}_{i,-} \approx -\hat{c}_i$ and $\Omega_{i,-} \approx \omega_{c,i}$.

As the traps BEC modes are due to the LP branches we rewrite $\hat{H}_{\text{int}}$ neglecting the other polariton branches as:

$$\hat{H}_{\text{int}} = -\hbar g_0\,\hat{p}_{2,-}^\dagger\hat{p}_{1,-}\hat{b}_m^\dagger + h.c. \tag{6}$$

with $g_0 = S_x\, g_0^{DP} + S_c\, g_0^{RP}$, where $S_x = \cos\theta_1 \cos\theta_2$ and $S_c = \sin\theta_1 \sin\theta_2$.

In what follows we work solely with the LP phonon branches omitting the subindex "-", and retaining the trap index $\{1, 2\}$. We incorporate a strong effective driving at $\omega_\alpha$ tuned to the blue-shifted polariton mode, of frequency $\Omega_1$, and a much weaker effective driving at $\omega_\beta$ tuned to feed the lower energy polariton mode of the neighbor trap, of frequency $\Omega_2$. We write the optomechanical interaction assuming a situation with $\Omega_1 - \Omega_2 \gtrsim \omega_m$, making neighbor trap mode mixing in the OM coupling –if nonzero as we assume here– relevant. The full Hamiltonian reads

$$\hat{H} = \hbar\omega_m \hat{b}_m^\dagger \hat{b}_m + \hbar\Omega_1 \hat{p}_1^\dagger \hat{p}_1 + \hbar\Omega_2 \hat{p}_2^\dagger \hat{p}_2 - (\hbar g_0\, \hat{p}_2^\dagger \hat{p}_1 \hat{b}_m^\dagger + h.c) \\ + i\hbar\zeta_{a,1} \left( \mathrm{e}^{-i\omega_\alpha t} \hat{p}_1^\dagger - \mathrm{e}^{i\omega_\alpha t} \hat{p}_1 \right) + i\hbar\zeta_2 \left( \mathrm{e}^{-i\omega_\beta t} \hat{p}_2^\dagger - \mathrm{e}^{i\omega_\beta t} \hat{p}_2 \right) + \hat{H}_{\mathrm{decay}}. \tag{7}$$

Cavity losses are included in $\hat{H}_{\mathrm{decay}}$ parametrized by the linewidths $\kappa_1$, $\kappa_2$ of the polariton traps, and the phonon linewidth $\Gamma_m$. The driving amplitudes are controlled by the rates $\zeta_1$ and $\zeta_2$.[15]

The customary approach of working in the interaction picture and linearizing the equations of motion around equilibrium (for classical complex amplitudes) leads to –after solving for the perturbations in Fourier space– the optomechanically modified phonon effective lifetime $\Gamma_{\mathrm{eff}} = \Gamma_m(1 - C)$. This means that the classically estimated threshold for self-oscillation is reached provided that

$$1 < C = 4 \frac{N_1 |g_0|^2}{\kappa_2 \Gamma_m}. \tag{8}$$

The latter result holds for $\Omega_2 = \omega_\beta$, and $\Omega_1 - \Omega_2 = \omega_m$; for the strongest driven trap mode the optomechanical coupling has been neglected: Under the undepleted pump approximation, at $\Omega_1 = \omega_\alpha$, this mode is assumed having a time-independent dominant population $N_1$.

We now focus on the experimental parameters at hand. First the number of polaritons in the BEC can be estimated as $N_1 = \eta \frac{P_{Pump}}{\hbar\omega} \tau$. Here $\eta$ is the fraction of excited electron-hole pairs that condense from the exciton reservoir into the BEC. $P_{Pump}$ is the non-resonant laser power, $\hbar\omega$ the laser photon energy, so that $\frac{P_{Pump}}{\hbar\omega}$ is the number of excitons generated by the non-resonant excitations. And $\tau$ is the exciton reservoir lifetime. From time-resolved differential reflectivity measurements, we estimate $\tau \sim 2\,\mathrm{ns}$. With this value we get $N_1 \sim \eta \times 10^7\, P_{Pump}$, with $P_{Pump}$ given in mW. Based on previous studies of cavity confined modes in semiconductor microcavities, we assume the mechanical $Q$-factor is $Q_m \sim 10^4$,[10–12] and thus $\Gamma_m = 2\pi\nu_0/Q_m \sim 2\pi \times 2$ MHz. We take as $\kappa_1 = \kappa_2$ the BEC decoherence rate, which from the measured coherence time of $\sim 530$ ps

($Q_{BEC} \sim 2\times10^5$) is $\kappa_i \sim 2\pi\times1.8$ GHz. The radiation pressure optomechanical coupling factor in a pillar microcavity of similar size as the studied traps has been calculated in Ref.[6]. In the BEC the photon fraction is very close to 1/2, and thus we take half of the reported value, $g_0^{RP} \sim 2\pi\times25$ kHz (see the discussion below in Supplementary Note 8). From the threshold condition $C = 1$ taken with $|g_0| = |g_0^{RP}|$ one thus obtains, based on the first-order optomechanical interaction Hamiltonian described in Supplementary Equation (7), that the self-oscillation threshold condition for the BEC is $P_{Th} = \frac{1}{\eta\times10^7}\frac{\kappa\Gamma_M}{4(g_0^{RP})^2} \sim \frac{0.7}{\eta}$[mW].

Detailed photoemission studies of Bose-Einstein polariton condensates show that, for non-resonant excitation, the occupancy of the BEC state is macroscopic, with initial reports in planar structures placing it around 40% (see e.g. Fig. 6.11 in the PhD Thesis of J. Kasprzak).[13] More recent investigations in 3D-confined micropillars show that the occupation fraction of the polariton condensate can reach $\sim$ 70%. For the estimation of the self-oscillation threshold power and to be consistent with these estimations, we take the smaller value so as not to underestimate the required optomechanical coupling strength. Consequently, assuming that 40% of the photoexcited electron-hole pairs end up populating the BEC, this estimation gives $P_{Th} \sim 0.4$ mW. This implies that the hybrid polariton BEC optomechanical system is already in conditions of self oscillation at the powers required for condensation (Bose-Einstein condensation is observed above $\sim$ 19 mW for the 1.6 $\mu$m traps under the experimental conditions used). Consequently, based on these considerations self-oscillation should be observed whenever the conditions for double resonance are satisfied, i.e. that neighbor traps are red-detuned from the BEC integer numbers of $\nu_m^0$, as is indeed experimentally observed.

The experimental results indicate that higher-order terms in the phonon operators could also be present in $\hat{H}_{\text{int}}$. For instance, a quadratic interaction of the form $(-\hbar G_2\hat{p}_2^\dagger\hat{p}_1(\hat{b}_m^\dagger)^2 + h.c)$ is likely to be the most important contribution when the traps are detuned by $\Omega_1 - \Omega_2 = 2\omega_m$: as these are the surviving BEC-trap-mixing terms proportional to the operator $\hat{x}^2 \propto (\hat{b}_m + \hat{b}_m^\dagger)^2$ within the applied rotating wave approximation. In general, for higher-order couplings, the conditions for achieving the threshold depend nontrivially of the state of the system: namely, of the number of cavity phonons and polaritons in each trap. For simplicity we explore here the threshold condition for the pure quadratic case, i.e., with $g_0 = 0$ and $G_2 \neq 0$. For this purpose we work in the interaction picture of with respect to $H_0 = \hbar\frac{\omega_\alpha-\omega_\beta}{2}\hat{b}_m^\dagger\hat{b}_m + \hbar\omega_\alpha\hat{p}_1^\dagger\hat{p}_1 + \hbar\omega_\beta\hat{p}_2^\dagger\hat{p}_2$ and the resulting Hamiltonian

becomes

$$
\begin{aligned}
\hat{H} = & \hbar\Delta_{\text{vib}}\hat{b}_m^\dagger\hat{b}_m + \hbar\Delta_1\hat{p}_1^\dagger\hat{p}_1 + \hbar\Delta_2\hat{p}_2^\dagger\hat{p}_2 \\
& -\hbar G_2\hat{p}_2^\dagger\hat{p}_1\left(\hat{b}_m^\dagger\right)^2 - \hbar G_2\hat{p}_1^\dagger\hat{p}_2\left(\hat{b}_m\right)^2 \\
& +i\hbar\zeta_1\left(\hat{p}_1^\dagger - \hat{p}_1\right) + i\hbar\zeta_2\left(\hat{p}_2^\dagger - \hat{p}_2\right) + \hat{H}_{\text{decay}}
\end{aligned} \tag{9}
$$

with the definitions of the detunings $\Delta_{\text{vib}} \equiv \omega_m - \frac{\omega_\alpha - \omega_\beta}{2}$, $\Delta_1 \equiv \Omega_1 - \omega_\alpha$, and $\Delta_2 \equiv \Omega_2 - \omega_\beta$, respectively. The equations of motion generated by this Hamiltonian are treated classically linearizing around equilibrium,[14,16] namely using $p_i(t) = p_{i,eq} + \delta p_i(t)$, $b_m(t) = b_{m,eq} + \delta b_m(t)$ and their conjugate field amplitudes. By specializing for the ideal detuning condition $\Delta_1 = \Delta_2 = \Delta_{\text{vib}} = 0$ we get

$$
\begin{aligned}
\delta\dot{p}_1 &= -\frac{\kappa_1}{2}\delta p_1 + i2G_2 p_{2,\text{eq}} b_{m,\text{eq}}\delta b_m + iG_2\delta p_2\left(b_{m,\text{eq}}\right)^2 \\
\delta\dot{p}_2 &= -\frac{\kappa_2}{2}\delta p_2 + i2G_2 p_{1,\text{eq}} b_{m,\text{eq}}^*\delta b_m^* + iG_2\delta p_1\left(b_{m,\text{eq}}^*\right)^2 \\
\delta\dot{b}_m &= -\frac{\Gamma_m}{2}\delta b_m + i2G_2\left(\delta p_{1,\text{eq}} p_2^* b_{m,\text{eq}}^* + p_{1,\text{eq}}\delta p_2^* b_{m,\text{eq}}^*\right. \\
& \quad \left. + p_{1,\text{eq}} p_{2,\text{eq}}^*\delta b_m^*\right)
\end{aligned} \tag{10}
$$

We solve the above system in Fourier space setting $\omega = 0$, as in this interaction picture it represents the behavior at the phonon frequency. We proceed to remove the polariton degrees of freedom in order to obtain the effective response of the phonon position, $\delta x \propto \delta b_m + \delta b_m^*$, to a noise perturbation $\xi_x$. After some algebraic steps one arrives to equation $\Sigma_0\,\delta b + \Sigma_1^*\,\delta b^* = \xi_x$ and its equivalent conjugated form, from where we get that $\delta x \propto (|\Sigma_0|^2 - |\Sigma_1|^2)^{-1}\xi_x$. The threshold to a nonlinear instability is achieved when $|\Sigma_0|^2 - |\Sigma_1|^2 = 0$, as $\delta x$ diverges for this situation. Consistently, the factor $|\Sigma_0|^2 - |\Sigma_1|^2$ also appears in the numerator of the effective phonon linewidth,

$$
\Gamma_{\text{eff}} = 2\Sigma_0(|\Sigma_0|^2 - |\Sigma_1|^2)/(|\Sigma_0|^2 + |\Sigma_1|^2)\ , \tag{11}
$$

and its reduction to zero marks the onset of self-oscillation. The obtained $\Sigma_i$ are defined as

$$
\Sigma_0 \equiv \frac{\Gamma_m}{2}\left(1 - \frac{16G_2^2 N_1 N_b}{\tilde{\kappa}_2\Gamma_m} + \frac{16G_2^2 N_2 N_b}{\tilde{\kappa}_1\Gamma_m}\right) \tag{12}
$$

$$
|\Sigma_1| \equiv 2G_2\sqrt{N_1 N_2}\left|1 - 2G_2^2 N_b^2\left(\frac{4}{\tilde{\kappa}_1\kappa_2} + \frac{4}{\kappa_1\tilde{\kappa}_2}\right)\right| \tag{13}
$$

where $\tilde{\kappa}_1 \equiv \kappa_1 + \frac{4G_2^2N_b^2}{\kappa_2}$, $\tilde{\kappa}_2 \equiv \kappa_2 + \frac{4G_2^2N_b^2}{\kappa_1}$ describes the *quadratic* broadening due to phonons of the polariton modes. Equations (3) and (4) reproduced in the main text correspond to the above Supplementary Equations (12) and (13) when $\kappa_1 = \kappa_2 = \kappa$ is assumed.

We have checked that for the latter parameters the lowest order in $G_2$ approximation to $|\Sigma_0|^2 - |\Sigma_1|^2$ works well for predicting the lowest value of $G_2$ required to observe the threshold compatible with our measurements. This approximation is contained in the following inequality

$$\frac{16G_2^2 N_1 N_2}{\Gamma_m^2} + \frac{32G_2^2 N_1 N_b}{\tilde{\kappa}_2 \Gamma_m} - \frac{32G_2^2 N_2 N_b}{\tilde{\kappa}_1 \Gamma_m} > 1. \tag{14}$$

We get that a coupling constant of value $G_2^{(\mathrm{Th})} = 2\pi \times 1.25\mathrm{Hz}$ would suffice to generate a self-oscillation threshold power of 10 mW taking $N_1 = 0.2 \times 10^7 P[\mathrm{mW}]$, $N_2 = N_1/100$ and $N_b = 10^5$: these are conservative parameters consistent with the observation. The estimated value $G_2^{(\mathrm{Th})}$ is feasible and results to be two orders of magnitude smaller than the one reported in Ref.[18].

## SUPPLEMENTARY NOTE 8: ON THE MAGNITUDE OF $g_0$.

The linear $g_0^{RP}$ referenced in the previous section was evaluated for a single trap. For the two-mode situation considered here, one should account for the overlap integrals between polariton modes located in neighbor traps. The penetration of the polariton ground states in the barriers, obtained from realistic calculations based on the known trap potential,[1] amounts to $\sim 1\mu$m for low excitation densities. The separation between traps in the studied array is $\sim 3.2\mu$m, and thus the overlap integral based on these trap ground state wavefunctions is indeed small. The experiments however clearly show that the penetration in the barriers changes with the excitation density. Indeed, Supplementary Fig. 7 (which corresponds to zone (1) in Fig. 3 of the main text) shows that under this high excitation, the extension of the fundamental state increases considerably and overlaps significantly with the neighbor traps.

Based on our experimental observations, we speculate that an enhanced tunnelling might be reflecting 1) a role of the polariton-polariton interactions, or alternatively 2) some involvement of the trap excited states. In fact, repulsive interactions may modify the polariton distribution within the traps,[19] as well as blue-shift the polariton levels, thus enhancing the tunnel coupling between neighboring traps. As for the trap excited states, their mixing between traps is significantly larger.

The above considerations bring into consideration also the role of reservoir effects in the observed phenomena. Firstly we note that the blue-shift of the neighbor traps, which is a fundamental ingredient to access the observed resonant detunings, seems to evidence a spatial extension of the reservoir that exceeds the intensity profile of the pump. This might be compatible with very interesting recent experiments performed in similar but unstructured GaAs microcavities, where

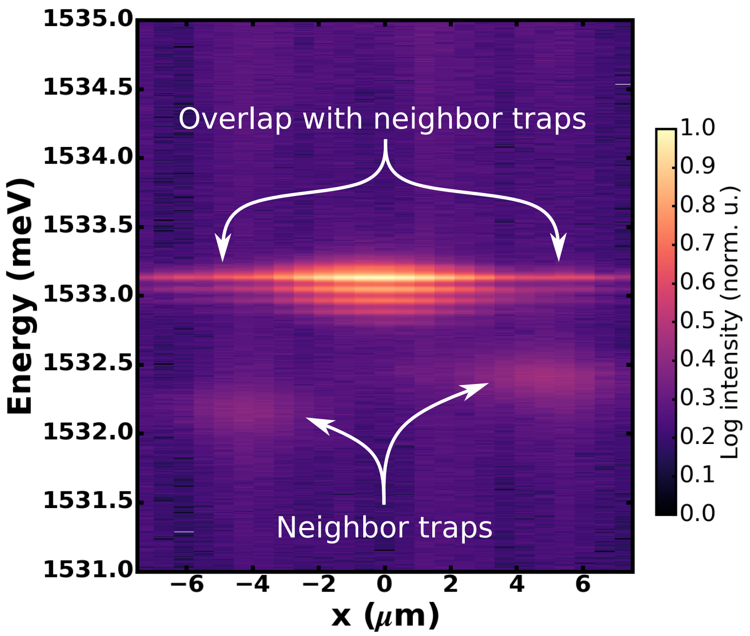


**Supplementary Figure 7. Spatial image of the emission of the 1.6$\mu$m trap array for the condition of phonon assisted PL.** The non-resonant *cw* excitation power corresponds to that described as (1) in Fig. 3 of the main text. Note the emission corresponding to the neighbor traps, and the large penetration of the ground polariton state of the pumped trap into the neighbor sites.

significant influence of the reservoir is found tens of micrometers away from the laser pump spot. These results have been interpreted as signalling an interaction-induced strongly enhanced transport of high-momentum excitonic polaritons away from the bottleneck region.[20]. And secondly, as mentioned in the previous paragraph, interactions might have a role in the efficient optomechanical mechanism evidenced in the experiments. Besides the possible repulsion-induced change of the polariton distribution in the trap array, we note that because the trap energies are determined by the exciton-exciton coupling between trap polaritons and reservoir excitons, we cannot a-priori exclude that a hitherto undiscovered optomechanical coupling mechanism involving excitonic correlations might be present.

Another relevant issue is a possible role of dissipative coupling in the observed phenomena.[21] It is our understanding that for the studied high-Q microcavities, with long-lived excitons, and with slightly negative detuning (larger photonic character), dispersive coupling should be playing the major role. We do not find evidence of a possible role of absorption. However, it cannot be exclude. Systematic studies of the phonon lasing characteristics in polariton trap structures of varying exciton-photon detuning might bring further insight on this issue.

Summing up then: the experimental results are clear in that an efficient polariton transfer

between traps involving the coupling with phonons is active in the studied system. These results also show that much is left to explore in the field of optomechanics of cavity polaritons, including a detailed microscopic physical description of the processes involved.

---

* Corresponding author, E-mail: afains@cab.cnea.gov.ar

## SUPPLEMENTARY REFERENCES


[1] Kuznetsov, A. S., Helgers, P. L., Biermann, K., and Santos, P. V. Quantum confinement of exciton-polaritons in a structured (Al, Ga) As microcavity. *Physical Review B* **97**, 195309 (2018).

[2] Rozas, G., Jusserand, B., and Fainstein, A. Fabry-Pérot-multichannel spectrometer tandem for ultra-high resolution Raman spectroscopy. *Rev. Sci. Instrum.* **85**, 013103 (2014).

[3] Johnson, S. G., Ibanescu, M., Skorobogatiy, M. A., Weisberg, O., Joannopoulos,J. D., and Fink, Y. Perturbation theory for Maxwell's equations with shifting material boundaries. *Phys. Rev. E* **65**, 066611 (2002).

[4] Ding, L., Baker, C., Senellart, P., Lemaître, Ducci, S., Leo, G., and Favero, I. High frequency GaAs nano-optomechanical disk resonator. *Phys. Rev. Lett.* **105**, 263903 (2010).

[5] Baker, C., Hease, W., Dac-Trung Nguyen, Andronico, A., Ducci, S., Leo, G., and Favero, I. Photoelastic coupling in gallium arsenide optomechanical disk resonators. *Opt. Express* **22**, 14072 (2014).

[6] Villafañe, V., Sesin, P., Soubelet, P., Anguiano, S., Bruchhausen, A. E., Rozas, G., Gomez Carbonell, C., Lemaître, A., and Fainstein, A. Optoelectronic forces with quantum wells for cavity optomechanics in GaAs/AlAs semiconductor microcavities. *Phys. Rev. B* **97**, 195306 (2018).

[7] Yariv, A., and Yeh, P., in Optical Waves in Crystals (Wiley, New York, 1984).

[8] Rytov, S. M. Acoustical properties of a thinly laminated medium. *Akust. Zh.* **2**, 71 (1956) [*Sov. Phys. Acoust.* **2**, 68 (1956)]

[9] Fainstein A. and Jusserand B. Raman Scattering in Resonant Cavities, in M. Cardona and R. Merlin (eds) *Light Scattering in Solid IX. Topics in Applied Physics*, vol. 108. Springer, Berlin, Heidelberg (2006).

[10] Anguiano, S., Bruchhausen, A. E., Jusserand, B., Favero, I., Lamberti, F. R., Lanco, L., Sagnes, I., Lemaitre, A., Lanzillotti-Kimura, N. D., Senellart, P., and Fainstein, A. Micropillar resonators for optomechanics in the extremely high 19–95-GHz frequency range. *Phys. Rev. Lett.* **18**, 263901 (2017).

[11] Lagoin, C., Perrin, B., Atkinson, P., Garcia-Sanchez, D. High spectral resolution of GaAs/AlAs phononic cavities by subharmonic resonant pump-probe excitation. *Phys. Rev. B* **99**, 060101 (2019).

[12] Machado, D.H.O., Crespo-Poveda, A., Kuznetsov, A. S., Biermann, K., Scalvi, L.V.A., and Santos, P. V. Generation and Propagation of Superhigh-Frequency Bulk Acoustic Waves in GaAs. *Phys. Rev. Applied* **12**, (2019).

[13] Kasprzak, J. Condensation of exciton polaritons. Condensed Matter [cond-mat]. Universit Joseph-Fourier - Grenoble I, 2006. English. tel-00118316.

[14] Aspelmeyer, M., Kippenberg, T.J., and Marquardt, F. Cavity optomechanics. *Rev. Mod. Phys.* **86**, 1391 (2014).

[15] For a source of frequency $\omega_\alpha$, power $P_\alpha$ and with the cavity $i$ being able to accept excitations from the source at the rate $\kappa_i^{\mathrm{ext}}$, one gets $|\zeta_i|^2 = \frac{P_\alpha \kappa_i^{\mathrm{ext}}}{\hbar\omega_\alpha}$.

[16] For a detailed discussion on the classical and quantum treatment of the optomechanical instability see

Ref.[17].

[17] Ludwig, M., Kubala, B., Marquardt, F. The optomechanical instability in the quantum regime. *New J. Phys.* **10**, 095013 (2008).

[18] Paraiso, T.K., Kalaee, M., Zang, L., Pfeifer, H., Marquardt, F., Painter, O. Position-squared coupling in a tunable photonic crystal optomechanical cavity. *Phys. Rev. X* **5**, 041024 (2015).

[19] Ferrier, L., Wertz, E., Johne, R., Solnyshkov, D.D, Senellart, P., Sagnes, I., Lemaître, A., Malpuech, A., and Bloch, J. Interactions in Confined Polariton Condensates. *Phys. Rev. Lett.* **106** (2011).

[20] See for example M. Boozarjmehr *et al*, arXiv:1912.07765 [cond-mat.quant-gas]), and references therein.

[21] Kyriienko, O., Liew, T. C. H., and Shelykh, I. A. Optomechanics with cavity polaritons: dissipative coupling and unconventional bistability. *Phys. Rev. Lett.* **112**, 076402 (2014).
