# Supplementary material for: Polariton-driven phonon laser
Source: Nat Commun. 2020 Sep 11;11:4552. doi: 10.1038/s41467-020-18358-z (PMC7486378; doi:10.1038/s41467-020-18358-z)
Supplement: Supplementary file 2 — Reporting Summary [file 41467_2020_18358_MOESM2_ESM.pdf]

natureresearch

Corresponding author(s): A. Fainstein

Lasing Reporting Summary

Nature Research wishes to improve the reproducibility of the work that we publish. This form is intended for publication with all accepted papers reporting claims of lasing and provides structure for consistency and transparency in reporting. Some list items might not apply to an individual manuscript, but all fields must be completed for clarity.

For further information on Nature Research policies, including our data availability policy, see Authors & Referees.

Experimental design

Please check: are the following details reported in the manuscript?

1. Threshold

Plots of device output power versus pump power over a wide range of values indicating a clear threshold

☐ Yes
☒ No

The excitation power in the reported experiments is used simultaneously 1) to increase the polariton population in the pumped trap to attain threshold, and 2) to tune the energy of the pumped trap polariton levels respect to the neighbor trap so that the conditions for the required two-mode resonance is attained. Because this later detuning is defined by excitonic correlations, the two features are intimately connected. The condition for the two-mode polariton resonances require their separation being an even integer repetitions of the confined phonon energy. Thus, the required detuning makes discrete steps from -6\omega_0, to -4\omega_0, and finally to -2\omega_0 (\omega_0 here is the energy of the confined phonon). At 6 \omega_0 the system is still below threshold, while at -4\omega_0 and -2\omega_0 it is above threshold for mechanical self-oscillation (phonon "lasing").

2. Linewidth narrowing

Plots of spectral power density for the emission at pump powers below, around, and above the lasing threshold, indicating a clear linewidth narrowing at threshold

☐ Yes
☒ No

Figures 2 and 3 display the evolution with excitation power of the device's photoluminescence emission. The abrupt line-width narrowing at the threshold characterizes the onset of the polariton Bose-Einstein condensation. The generated phonons are evidenced through their modulation of the polariton BEC energies, which are broader than those of the phonons (by more than an order of magnitude). Thus, the observed linewidths reflect properties of the BEC and not of the coherently self-oscillating phonons.

Resolution of the spectrometer used to make spectral measurements

☒ Yes
☐ No

This information can be found both in the main text: on Section "Results" subsections "Optomechanically induced amplification" and "Mechanical self-oscillations", including reference to a paper where the methods are explained in detail, and in the section "Methods". A detailed description can also be found in the provided Supplementary Note 2.

3. Coherent emission

Measurements of the coherence and/or polarization of the emission

☒ Yes
☐ No

This is demonstrated in the experiments displayed in Figs. 3 and 4, and through the analysis based on Eq. (1). The system evolves from enhanced emission of incoherent phonons (region defined as "1" in Fig. 3), to a fully coherent situation characterized by the emergence of symmetric well resolved side-bands in the BEC emission (regions "2" and "3"). These resolved side-bands demonstrate the existence of a coherent mechanical self-oscillation (phonon "lasing"), and their analysis provides quantitative information on the amplitude of the coherent vibration, and consequently on the number of phonons in this coherent state (details can be found in the Supplementary Notes 4 and 5).

4. Beam spatial profile

Image and/or measurement of the spatial shape and profile of the emission, showing a well-defined beam above threshold

☒ Yes
☐ No

Figure 2(panel c) shows the spatial profile of the well resolved phononic side-bands.

5. Operating conditions

Description of the laser and pumping conditions
Continuous-wave, pulsed, temperature of operation

☒ Yes
☐ No

The "phonon laser" was demonstrated for continuous-wave non-resonant laser excitation, operating at low temperatures (5K). This information can be found in the description of the results in Figs. 2 and 3, and in of the provided Supplementary Note 2.

Threshold values provided as density values (e.g. W cm^{-2} or J cm^{-2}) taking into account the area of the device

☐ Yes
☒ No

As stated in point (1) above, we know that threshold for phonon "lasing" occurs somewhere between regions "1" and "2" in Fig. 3. The experimental laser powers used are given in Fig. 2 in relation to the threshold for polariton Bose-Einstein condensation (P_Th). The value of P_Th ~ 19 mW and the laser spot size used (~3 µm) are provided in this same Fig. 2.

6. Alternative explanations

Reasoning as to why alternative explanations have been ruled out as responsible for the emission characteristics
e.g. amplified spontaneous, directional scattering; modification of fluorescence spectrum by the cavity

☒ Yes
☐ No

A detailed analysis of the characteristics of the observed emission is provided, distinguishing what arises from emission of different traps, and what corresponds to side-bands of the BEC emission of the pumped trap. The spatial images allow to distinguish these two possible sources (Fig. 2 of the main text). The energy of the side-bands on the other hand correspond precisely with the energy of the confined phonon vibration, as discussed in detail in the Supplementary Notes 7.

7. Theoretical analysis

Theoretical analysis that ensures that the experimental values measured are realistic and reasonable
e.g. laser threshold, linewidth, cavity gain-loss, efficiency

☒ Yes
☐ No

A theoretical analysis of the cavity optomechanical problem is in section "Results" subsection "Mechanical self-oscillation", and continues in subsection "Optomechanical model with linear and quadratic coupling". The discussion starts analyzing our results using the existing cavity-optomechanical models. In addition, we include a theoretical analysis of quadratic terms in the optomechanical interaction in the context of resonant two-polariton modes, something that, to the best of our knowledge, has not been previously considered. A detailed description related to this discussion, and to the intervening interactions and parameters, is also included in the Supplementary Notes 7 and 8.

8. Statistics

Number of devices fabricated and tested

☒ Yes
☐ No

This information can be found briefly in the main text in section "Results", and is treated with more detail in the provided Supplementary Note 1. The structures correspond to the same reported for the study of Bose-Einstein condensation in traps in our Ref.[26]. The technology to grow and fabricate these devices is established in this latter paper and references therein. This first investigation of the new reported phenomena concentrates in one of these devices and trap arrays.

Statistical analysis of the device performance and lifetime (time to failure)

☐ Yes
☒ No

We are reporting a novel phenomenon related to the coherent mechanical self-oscillation induced by the strong optomechanical coupling in the context of trapped exciton-polariton Bose-Einstein condensates. As stated by one of the referees, "the phenomenon is quite new and important for developing new direction in optomechanics field". While the present level of development already demonstrates very efficient phonon "lasing", clearly many paths are open for future developments and performance optimization.
